# Protective Efficacy of Two Novel DNA Vaccine Candidates Encoding TgGRA28 and TgGRA83 with an IL-28B Molecular Adjuvant Against Acute and Chronic Toxoplasmosis in Mice

**DOI:** 10.3390/vaccines13121180

**Published:** 2025-11-21

**Authors:** Jun Fang, Jingqi Mu, Rui Li, Jia Chen

**Affiliations:** Department of Radiology, The Affiliated People’s Hospital of Ningbo University, Ningbo 315040, China

**Keywords:** *Toxoplasma gondii*, IL-28B, TgGRA28, TgGRA83, DNA vaccine, protective immunity

## Abstract

Background: *Toxoplasma gondii* is a globally distributed apicomplexan parasite capable of causing congenital infections and spontaneous abortions in humans. While the parasite-secreted effector proteins TgGRA28 and TgGRA83 are known to mediate virulence or immune modulation, their potential as vaccine targets remains unexplored. Despite its immunomodulatory properties, the role of IL-28B (a type III interferon) in enhancing DNA vaccine efficacy against *T. gondii* infection remains unclear. Methods: In this study, we constructed eukaryotic expression plasmids pVAX-GRA28, pVAX- GRA83 and pVAX-IL-28B. After transfection into -293-T cell, protein expression encoding TgGRA28 and TgGRA83 was confirmed via indirect immunofluorescence assay (IFA), while IL-28B expression was analyzed by ELISA. Subsequently, C57BL/6J or IFNαR1 knockout mice were immunized with single or dual-antigen DNA vaccines, with or without the molecular adjuvant pVAX-IL-28B. Immune responses were assessed through *Toxoplasma*-specific antibody levels, cytotoxic T lymphocyte (CTL) activity, cytokine profiling (IFN-γ, IL-2, IL-12p40, IL-12p70), and flow cytometric analysis of lymphocyte subsets and dendritic cells (DCs). Protective efficacy was determined by survival rates and brain cyst burden following challenge with 100 or 10 ME49 *T. gondii cysts*, respectively. Results: Vaccination with pVAX-GRA28 and pVAX-GRA83 elicited robust humoral immune responses with increased *T. gondii*-specific IgG levels and also Th1-polarized immunity, characterized by elevated IgG2a/IgG1 ratio, IFN-γ-dominant cytokine responses, and enhanced DCs, CD4+ and CD8+ T-cell activation. The cocktail vaccine conferred superior protection compared to single-antigen formulations, significantly improving survival and reducing cyst formation. Co-administration of pVAX-IL-28B further augmented vaccine-induced immunity, enhancing both cellular and humoral responses. Moreover, these DNA immunization with pVAX-GRA28 and pVAX-GRA83 plus pVAX-IL-28B induced robust protective immunity that was largely independent of type I IFN signaling, consistent with type III IFN biology. Conclusions: Our findings demonstrate that TgGRA28 and TgGRA83 are promising vaccine candidates against toxoplasmosis, capable of inducing protective immunity against acute and chronic infection. Moreover, IL-28B serves as a potent genetic adjuvant, warranting further investigation for its broader application in vaccines targeting apicomplexan parasites.

## 1. Introduction

*Toxoplasma gondii*, an obligate intracellular apicomplexan parasite, exhibits remarkable host plasticity, infecting diverse warm-blooded vertebrates including domesticated and wild species [1]. With seroprevalence rates approaching 30% in some human populations, this zoonotic pathogen imposes substantial global health burdens across species barriers [2]. Transmission occurs primarily through ingestion of sporulated oocysts contaminating horticultural products and water sources, or via consumption of undercooked meat containing bradyzoite cysts [3]. While immunocompetent hosts typically maintain asymptomatic infection, the parasite demonstrates particular clinical virulence in vulnerable populations, causing fetal neuropathogenesis and spontaneous abortion during gestational exposure [4], life-threatening encephalitis in HIV/AIDS patients [5], and severe complications in iatrogenically immunosuppressed individuals [6]. The agricultural impact is equally profound, with *T. gondii*-induced reproductive failure in small ruminants generating significant economic losses in livestock production systems [7].

While current chemotherapeutic regimens (e.g., pyrimethamine-sulfadiazine combination therapy and spiramycin) demonstrate efficacy against acute toxoplasmosis, their therapeutic limitations are profound—failing to eradicate persistent tissue cysts during chronic infection [8]. This therapeutic gap underscores the critical need for immunoprophylactic interventions, positioning vaccine development as a paramount public health priority for the control of *T. gondii* infection [9]. Although the live-attenuated S48 strain vaccine (Toxovax^®^) has proven efficacious in preventing ovine abortion storms, its zoonotic potential risks and side effects preclude application in human medicine or food animal production [10]. Consequently, the development of next-generation vaccines exhibiting cross-species protective efficacy remains an urgent unmet medical need [11]. Substantial advances have been achieved across multiple vaccine platforms, including whole-parasite approaches (inactivated/attenuated strains), molecular vaccines (DNA plasmids, recombinant subunits), multi-antigen formulations (subunit vaccines) [12]. Also, cutting-edge research prioritizes the identification of immunodominant antigens capable of eliciting sterilizing immunity, with particular focus on invasion-associated surface antigens (SAG family proteins), micronemal secretion system components (MICs), dense granule effectors (GRAs), and rhoptry virulence determinants (ROPs) [9,13].

Among characterized *T. gondii* antigens, the effector proteins TgGRA28 and TgGRA83 emerge as particularly promising vaccine candidates due to their unique mechanistic roles in host–pathogen interactions. The parasite effector GRA28, secreted via the *T. gondii* MYR1-dependent protein export pathway, translocates to the host nucleus where it hijacks endogenous chromatin remodeling complexes. This molecular subversion reprograms host transcriptional networks to induce chemotactic migration of infected macrophages—a critical mechanism for systemic parasite dissemination, suggesting that TgGRA28 promotes parasite dissemination by inducing dendritic cell-like migratory properties in infected macrophages [14]. Additionally, the *T. gondii* GRA83 has been characterized to be a novel effector modulating the host’s innate immune response to regulate parasite infection [15]. Despite their established roles in virulence or immune modulation, the capacity of TgGRA28/TgGRA83 to elicit protective immunity remains a fundamental unanswered question in *Toxoplasma* vaccinology.

Adjuvants play a pivotal role in modulating vaccine-induced immunity, both amplifying the magnitude and polarizing the quality of adaptive responses [16]. Contemporary adjuvant research has increasingly focused on cytokine-based strategies, with particular emphasis on: (i) pleiotropic cytokines (IL-21/IL-15) to enhance memory formation, (ii) homeostatic cytokines (IL-7/IL-15) to sustain lymphocyte populations, and (iii) alarmin cytokines (IL-33) to potentiate mucosal immunity [17,18]. These molecular adjuvants demonstrate exceptional promise for DNA vaccine platforms, where they can concomitantly broaden immunologic coverage and enhance both cellular (Th1/CTL) and humoral responses through targeted immunomodulation [19,20,21]. These findings suggest that cytokines could be strategically employed as genetic adjuvants to simultaneously enhance the magnitude and breadth of adaptive immunity—encompassing both humoral and cellular responses—in DNA vaccine development. Despite the therapeutic promise of cytokines including IL-33 and IL-15, their clinical translation remains constrained by dose-limiting toxicities [20,21]. So, cutting-edge strategies in anti-*Toxoplasma* vaccine research now prioritize the systematic screening of immunomodulatory cytokines as molecular adjuvants to overcome current limitations in sterilizing immunity and long-term protection [22]. IL-28B (IFN-λ3), a type III interferon family member, exhibits pleiotropic biological activities encompassing antiviral defense, immunomodulation, and tumor suppression [23]. Previous studies have elucidated its capacity to modulate regulatory T cell (Treg) responses in vaccination contexts. When employed as a DNA vaccine adjuvant, IL-28B was shown to enhance adaptive immunity while concurrently suppressing splenic Treg populations [24]. Another work extended these findings to tuberculosis subunit vaccination, demonstrating consistent Treg downregulation [25]. However, the effect of IL-28B on the DNA vaccine candidates against *T. gondii* remain unclear.

This study systematically evaluates the immunoprotective efficacy of pVAX-GRA28 and pVAX-GRA83 DNA vaccines against *T. gondii* infection in murine models. Furthermore, we engineered a novel pVAX-IL-28B expression construct and investigated its adjuvant potential when co-administered with bivalent TgGRA28/TgGRA83 DNA vaccine formulations.

## 2. Materials and Methods

### 2.1. Animals

Female outbred Kunming mice (6–8 weeks old, specific-pathogen-free) were obtained from Zhejiang Laboratory Animal Center, and inbred C57BL/6J and IFNαR1 knockout mice were purchased from Beijing Vital River Laboratory Animal Technology Co., Ltd. (Beijing, China), with all animals conducted in the Animal Ethics Procedures and Guidelines of the People’s Republic of China. Mice were acclimatized for ≥7 days under controlled conditions (22 ± 1 °C, 55 ± 5% humidity, 12/12 h light/dark cycle) with ad libitum access to autoclaved food and UV-treated water. All animal procedures were approved by the Animal Research Ethics Committee of Ningbo University (Approval No. SYXK(ZHE)2019-0005).

### 2.2. Cells, Parasites and Antigens

*T. gondii* ME49 strain tachyzoites were resuscitated from liquid nitrogen storage and maintained in vitro culture, while ME49 strain cysts were harvested from Kunming mouse brains at 30 days post-oral inoculation with 10 cysts [26]. Concurrently, 293-T cells were cultured in DMEM (Invitrogen, Carlsbad, CA, USA) supplemented with 10% heat-inactivated FBS and antibiotics (100 IU/mL penicillin-streptomycin) at 37 °C with 5% CO_2_. For *Toxoplasma* lysate antigen (TLA) preparation, ME49 strain tachyzoites were suspended in PBS (10 mM sodium phosphate, 0.15 M NaCl, pH 7.2), subjected to ultrasonic disruption (Sigma, St. Louis, MO, USA), and centrifuged (2100× *g*, 15 min, 4 °C). The supernatant was sterile-filtered (0.2 μm, Sigma), quantified spectrophotometrically (Eppendorf, Hamburg, Germany), and stored at −80 °C as TLA stock.

### 2.3. Construction of the Eukaryotic Expression Plasmids

To generate pVAX I-based plasmids expressing IL-28B (The open reading frame of murine IL-28B, NCBI Reference Sequence: XM_006540130.2, spanning 746 base pairs and including the signal peptide-coding sequence, was cloned into the pVAX1 vector.), TgGRA28 (The open reading frame of murine GRA28, NCBI Reference Sequence: XM_002368026.1, spanning 2100 base pairs and including the signal peptide-coding sequence, was cloned into the pVAX1 vector.), or TgGRA83 (The open reading frame of murine GRA83, NCBI Reference Sequence: XM_002368026.1, spanning 2291 base pairs and including the signal peptide-coding sequence, was cloned into the pVAX1 vector.), target gene fragments were amplified by polymerase chain reaction (PCR) using cDNA templates derived from *T. gondii* ME49 strain tachyzoites or total RNA isolated from spleens of Kunming mice [17,19,25]. The following primer pairs, containing engineered restriction sites, were used: TgGRA28: forward 5′-GGGGTACCATGATCTGGCAGCTGCATTAT-3′ (Kpn I), reverse 5′-GCTCTAGAAGCTACTGAAATCAGTCAG-3′ (Xba I); TgGRA83: forward 5′-GGGGTACCATGGCGACTCAGTTAGCGCAT-3′ (Kpn I), reverse 5′-GCTCTAGAATCGGATACGATGCATTCAG-3′ (Xba I); IL-28B: forward 5′-CCGGAATTCATGACTGACCTCAGTACGTGACT-3′ (EcoR I), reverse 5′-GCTCTAGAACTAGCATGCCATGGGCATAA-3′ (Xba I). The PCR products were cloned into the pMD-18 T Vector (TaKaRa, Beijing, China), yielding intermediate constructs pMD-IL-28B, pMD-GRA28, and pMD-GRA83. Each insert was subsequently released by digestion with Kpn I and Xba I and ligated into the corresponding sites of the pVAX I vector (Invitrogen) using T4 DNA ligase, resulting in the final expression plasmids pVAX-IL-28B, pVAX-GRA28, and pVAX-GRA83. All constructs were verified by PCR and double restriction enzyme digestion.

He recombinant plasmids (pVAX-GRA28, pVAX-GRA83, pVAX-IL-28B, and the empty pVAX1 vector) were transformed into E. coli DH5α and purified using the Plasmid Mini Preparation Kit (Beyotime, Shanghai, China) according to the manufacturer’s instructions. The purified DNA was dissolved in TE. DNA quality was assessed by measuring the A260/A280 ratio using a spectrophotometer, with all preparations yielding ratios between 1.8 and 2.0. Endotoxin levels were determined by the Chromogenic LAL Endotoxin Assay Kit (Beyotime, Shanghai, China) and were confirmed to be below 0.1 EU/mg DNA.

### 2.4. In Vitro Expression Analysis of Recombinant Plasmids

For heterologous protein expression, 293-T cells were transfected with either pVAX-GRA28, pVAX-GRA83, or pVAX-IL-28B expression constructs, using empty pVAX I vector as negative control. Transfections were performed using Lipofectamine™ 2000 transfection reagent (Invitrogen, Carlsbad, CA, USA) following standard protocols. At 48 h post-transfection, cells were processed for immunofluorescence analysis as follows: (1) fixation with ice-cold 100% acetone for 30 min at −20 °C; (2) permeabilization with 0.1% Triton X-100 in PBS (PBST); (3) blocking with 5% BSA in PBST; (4) sequential incubation with primary antibody (goat anti-*T. gondii* polyclonal antiserum, 1:50 dilution in PBST) and secondary antibody (FITC-conjugated donkey anti-goat IgG, 1:1000 dilution; Proteintech Group Inc., Chicago, IL, USA); and (5) fluorescence imaging using a Zeiss Axioplan microscope equipped with appropriate filter sets (Carl Zeiss, Oberkochen, Germany).

For in vitro IL-28B expression analysis, 293-T cells were transfected with pVAX-IL-28B (experimental group) or empty vector (negative control) using Lipofectamine™ 2000 (Invitrogen, Carlsbad, CA, USA). After 48 h, culture supernatants were collected, and IL-28B secretion was quantified using a commercial ELISA kit (Abcam, Cambridge, UK) as per the manufacturer’s guidelines [21].

For in vivo IL-28B expression analysis, sera samples obtained from mice three days after the first immunization with pVAX-IL-28B or the empty pVAX I vector, and then IL-28B secretion was quantified using a commercial ELISA kit (Abcam, Cambridge, UK) as per the manufacturer’s guidelines [21].

To further detect specific expression of pVAX-GRA28/pVAX-GRA83, protein ex-pression of GRA28/GRA83 was analyzed by Western blot. The expression of GAPDH in these cell lysates served as internal controls. Briefly, transfected 293-T cells were lysed through five freeze–thaw cycles, and the resulting lysates were separated by SDS-PAGE. The resolved proteins were then electrophoretically transferred onto a nitrocellulose (NC) membrane (Sigma, St. Louis, MO, USA). Subsequently, the membrane was blocked with 5% bovine serum albumin (BSA) in PBST (PBS containing 0.05% Tween-20) for 1 h at room temperature to prevent non-specific binding. After blocking, the membrane was incubated for 1 h at room temperature with mouse sera (diluted 1:500) obtained two weeks after the third immunization with pVAX-GRA28/pVAX-GRA83. Following three washes with PBST, the membrane was probed with a horseradish peroxidase (HRP)-conjugated goat anti-mouse IgG secondary antibody (Sigma, USA; diluted 1:3000) for 1 h at room temperature. After an-other series of washes, immunoreactive bands were visualized using the Clarity™ Western ECL Blotting Substrate (Bio-Rad, Hercules, CA, USA) according to the manufacturer’s instructions. Signal intensity was quantified by densitometry using ImageJ software (version 1.53t; National Institutes of Health, Bethesda, MD, USA). Sera from PBS-injected mice served as the negative control.

### 2.5. Longitudinal Analysis of Antigen-Specific Humoral Immunity

To characterize the vaccine-induced antibody responses, serial serum samples were collected from immunized mice at 2-week intervals over a 6-week period and analyzed by ELISA using the SBA Clonotyping System-HRP Kit (Southern Biotech Co., Ltd., Birmingham, UK) as previously described [26]. Briefly, 96-well plates were coated with 10 μg/mL TLA in PBS (100 μL/well) overnight at 4 °C, followed by blocking with 1% BSA in PBS for 1 h at 37 °C. After three washes with PBST (PBS containing 0.05% Tween 20), plates were incubated with serially diluted serum samples (1 h, RT) and subsequently probed with HRP-conjugated anti-mouse IgG, IgG1, IgG2a, IgG2b, or IgG3 antibodies for subclass differentiation. Following additional washes, antibody binding was detected using ABTS peroxidase substrate (1.5% ABTS in citrate buffer, pH 4.0, containing 0.03% H_2_O_2_) with 30 min incubation at room temperature. Optical density at 450 nm was measured in triplicate using a Bio-TekEL × 800 microplate reader (Bio-TekEL × 800, Burlington, VT, USA), with blank-subtracted values used for quantitative analysis of antigen-specific antibody levels.

### 2.6. Evaluation of Cellular Immune Responses by Lymphoproliferation Assay

To assess antigen-specific T cell responses, splenocytes were isolated from immunized mice two weeks post-final immunization. Following mechanical dissociation through a 70-μm cell strainer and erythrocyte lysis using RBC lysis solution (Sigma, Livonia, MI, USA), lymphocytes were plated in triplicate (2 × 10^5^ cells/well) in complete RPMI 1640 medium (10% heat-inactivated FBS, 100 U/mL penicillin, 100 μg/mL streptomycin, 2 mM L-glutamine). Cells were stimulated with: (i) TLA (10 μg/mL), (ii) ConA (5 μg/mL; positive control; Sigma), or (iii) medium alone (negative control) for 72 h at 37 °C in a humidified 5% CO_2_ incubator. Proliferation was measured by MTT assay (10 µL of 3-(4,5-dimethylthiazol-2-yl)-2,5-diphenyltetrazolium bromide (MTT, Sigma)); 5 mg/mL, 4 h incubation) according to our previously described methods [26], with absorbance read at 570 nm using a microplate reader. The stimulation index (SI) was calculated as: SI = (OD570[TLA]/OD570[medium]): (OD570[ConA]/OD570[medium]). All experimental conditions included five independent experiments, with background absorbance from unstimulated controls subtracted from test values.

### 2.7. Quantification of Antigen-Specific Cytotoxic T Lymphocyte Responses

Cytotoxic T lymphocyte (CTL) activity was assessed using an LDH release assay (CytoTox 96^®^; Promega, Madison, WI, USA). Splenic lymphocytes isolated from immunized mice were stimulated with 100 U/mL recombinant murine IL-12 (eBioscience, San Diego, CA, USA) for 5 days to generate effector cells. For target cell preparation, EL-4 cells were transfected with eukaryotic expression plasmids using Lipofectamine™ 2000 (Invitrogen, Carlsbad, CA, USA). Cytolytic activity was measured by co-culturing effector and target cells at varying E:T ratios (10:1, 20:1, 40:1, and 80:1) for 6 h under standard culture conditions (37 °C, 5% CO_2_). Control wells included: (1) effector cells alone (effector spontaneous release), (2) target cells alone (target spontaneous release), and (3) lysed target cells (target maximum release). Specific cytotoxicity was calculated using the formula: [(Experimental LDH release − Effector spontaneous − Target spontaneous)/(Target maximum − Target spontaneous)] × 100. Data analysis was performed using results from five independent experiments.

### 2.8. Flow Cytometric Analysis of Splenic T Cell Subpopulations and DC Activations

Single-cell suspensions were prepared from spleens of experimental mice including female C57BL/6J mice and IFNαR1 knockout mice and subjected to comprehensive immunophenotyping of the percentages of CD4+ and CD8+ T lymphocytes. Briefly, splenocytes (5 × 10^5^ cells/mL) were stained with a fluorescent antibody panel containing phycoerythrin (PE)-conjugated anti-CD3, allophycocyanin APC-conjugated anti-CD4, and fluorescein isohiocyanate (FITC)-conjugated anti-CD8α (all from eBioscience, San Diego, CA, USA) for 30 min at 4 °C under light-protected conditions. Following two washes with ice-cold PBS, cells were fixed in 2% paraformaldehyde and resuspended in FACScan buffer (PBS supplemented with 1% FBS and 0.1% sodium azide).

For comprehensive evaluation of dendritic cell activation status, splenocytes from immunized mice including female C57BL/6J and IFNαR1 knockout mice were also subjected to multiparameter flow cytometric analysis. Cells were stained in parallel with four antibody panels containing: (i) CD11c-FITC (eBioscience, USA) paired with CD83-PE (clone Michel-19, BioLegend #121507), (ii) CD86-PE (eBioscience, USA), (iii) MHC class I-PE (eBioscience, USA), or (iv) MHC class II-PE (eBioscience, USA). All staining were performed for 30 min at 4 °C in the dark using titrated antibody concentrations, followed by two washes with ice-cold FACS buffer (PBS + 1% FBS + 0.1% sodium azide). All samples were acquired on a BD FACSCanto II flow cytometer (BD Biosciences) configured with 488 nm and 633 nm lasers, collecting a minimum of 50,000 viable lymphocyte-gated events per sample. Data analysis was performed using FlowJo software (version 10.8; BD Biosciences, Franklin Lakes, NJ, USA). Data analysis was performed using results from five independent experiments.

### 2.9. Quantification of Antigen-Specific Cytokine Production and Cytokine-Related Transcription Factors

To characterize the polarization and magnitude of antigen-specific immune responses, we performed cytokine analysis using splenocyte cultures from immunized mice by ELISA according to our previously described methods [26]. In brief, splenocytes were seeded at 2 × 10^5^ cells/well in complete RPMI-1640 medium and stimulated under two conditions, including TLA (10 μg/mL) and media control (negative control). Supernatants were collected at optimized time points: 24h for early-phase cytokines (IL-2, IL-4 and IL-12p40), 72 h for regulatory IL-10, and 96 h for effector cytokines (IFN-γ, IL-12p70). Cytokine quantification was performed using commercial ELISA kits according to the manufacturer’s instructions (Biolegend, San Diego, CA, USA) and reference to standard curves constructed with known amounts of these mouse recombinant cytokines. Data analysis was performed using results from five independent experiments.

To elucidate the transcriptional regulation of immune responses, we conducted quantitative analysis of key immune-regulatory transcription factors (p65/NF-κB, IRF8, and T-bet) through a rigorous qRT-PCR workflow. Total RNA was isolated from pooled splenocytes (*n* = 3 biologically independent samples per group) using TRIzol™ Reagent (Invitrogen), with RNA integrity verified by spectrophotometry (A260/A280 ratio >1.9) and microfluidic analysis (RNA Integrity Number ≥ 8.0, Agilent 2100 Bioanalyzer). Following DNase I treatment, 1 μg of high-quality RNA was reverse transcribed using GoScript™ Reverse Transcriptase (Promega Promega, Madison, WI, USA) with oligo(dT)18 primers in a 20 μL reaction volume. Quantitative PCR amplification was performed in technical triplicates on a LightCycler^®^ 480 System II (Roche, Basel, Switzerland) using SYBR Green I chemistry, with the following reaction conditions: 5 min at 95 °C for initial denaturation, followed by 45 cycles of 95 °C for 10 s, 60 °C for 30 s, and 72 °C for 20 s, with subsequent melt curve analysis (65–95 °C with 0.5 °C increments). Gene-specific primer pairs (Table 1) demonstrated amplification efficiency as determined by standard curve analysis.

### 2.10. Experimental Design and Immunological Evaluation of DNA Vaccination Against T. gondii

A cohort of 248 female C57BL/6J mice (6–8 weeks old) were randomly distributed into 8 experimental groups (*n* = 31/group) for comprehensive vaccine evaluation, with 120 allocated to immune response analysis and 128 to challenge experiments.

The immunization protocol consisted of three intramuscular administrations at 2-week intervals (weeks 0, 2, and 4) with: (i) 100 μg of the eukaryotic expression plasmid DNA (pVAX-GRA28, pVAX-GRA83, pVAX-IL-28B, pVAX-GRA28 + pVAX-GRA83, or pVAX-IL-28B + pVAX-GRA28 + pVAX-GRA83) in 100 μL sterile PBS; (ii) 100 μg empty pVAX vector (negative control); or (iii) PBS alone (vehicle control), with an additional untreated naive group serving as blank control.

The DNA vaccine dose (100 μg per mouse) was selected based on established protocols in the field and was well-tolerated, with no signs of toxicity observed in any of the immunized mice [27,28].

Serial serum samples were collected via tail vein puncture prior to each immunization and stored at −20 °C for humoral response analysis.

Fourteen days post-final immunization, animals were subjected to parallel evaluations: (1) A total of 15 mice per group were sacrificed for cellular immune assessments, including splenocyte profiling by flow cytometry (*n* = 5), CTL activity measurement (*n* = 5), and lymphoproliferation/cytokine production analysis (*n* = 5). The total number of biological replicates (used for the analysis of immune responses) was *n* = 15 per group. These mice were processed across five independent experimental cohorts (batches), with *n* = 5 mice per group in each group.

(2) Vaccine efficacy was evaluated in parallel challenge models two weeks post-immunization. Vaccine efficacy was evaluated in parallel challenge models two weeks post-immunization. To evaluate vaccine-induced protection against acute infection, mice (*n* = 10/group) were orally challenged with 100 cysts of the ME49 strain and monitored for survival over 60 days. The use of a moderate challenge dose with a low-to-moderate virulence strain represents a well-established model for survival analysis, as it enables a discerning evaluation of vaccine efficacy. This approach is particularly relevant because a high-dose challenge with the lethal RH strain can rapidly overwhelm suboptimal immunity, truncating survival times and potentially obscuring partial protection. Consequently, administering 80–100 cysts of the low-virulence PRU or ME49 strain has been adopted as a more reasonable protocol for assessing survival outcomes in vaccine studies [13,16]. Concurrently, the ability to control chronic infection was assessed by oral inoculation with 20 PRU cysts (*n* = 6/group), recapitulating the natural route of infection, followed by brain cyst quantification at 4 weeks [21,29]. This integrated design enabled simultaneous evaluation of vaccine-induced protection against both acute and chronic toxoplasmosis while characterizing underlying immune mechanisms. In addition, IFNαR1 knockout mice was used also for evaluation of vaccine-induced protection against both acute and chronic toxoplasmosis and splenocyte profiling by flow cytometry according the methods mentioned above.

### 2.11. Statistical Analysis

All data were analyzed using GraphPad Prism 9.0 (GraphPad Software) and SPSS Statistics 25 (IBM). Continuous variables (antibody titers, proliferation indices, cytokine concentrations) were compared using repeated-measures ANOVA with Holm-Šidák’s post hoc test for multiple com-parisons. Survival data were analyzed by Kaplan–Meier methodology with log-rank (Mantel–Cox) test for group comparisons. Normality was assessed using Shapiro–Wilk tests, and homogeneity of variance was verified by Brown-Forsythe test. For non-normally distributed data, Kruskal–Wallis test with Dunn’s correction was applied. All tests were two-tailed, with statistical significance defined as *p* < 0.05.

## 3. Results

### 3.1. Identification of Plasmids

**Figure 1:**
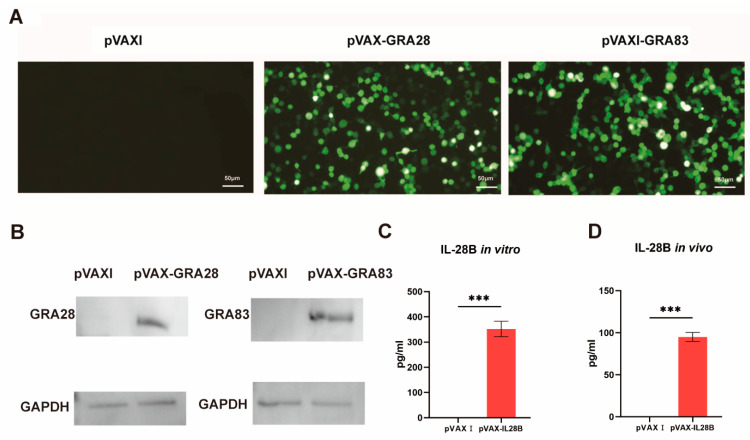
Determination of the expression of pVAX-GRA28, pVAX-GRA83 and pVAX-IL-24 in vitro. (**A**) Detection of recombinant TgGRA28 and TgGRA83 proteins expressed in transfected 293-T cells. Immunofluorescence analysis shows protein expression following transfection with pVAX-GRA28, pVAX-GRA83, or empty pVAX I vector control. (**B**) Western blotting analysis of the expression of GRA28 and GRA83 in 293-T cell lysates and empty pVAX I. (**C**) ELISA detection of pVAX-IL-28B expression in transfected 293-T cells. Cells were transfected with either pVAX-IL-28B or empty pVAX I vector control. (**D**) ELISA detection of in vivo IL-28B expression in blood samples collected from mice three days after the primary immunization with either pVAX-IL-28B or the empty pVAX1 vector. *** *p* < 0.001. Data are presented as the means ± SD.

To validate successful plasmid construction, 293-T cells transfected with either pVAX-GRA28 or pVAX-GRA83 exhibited distinct green fluorescence, whereas cells transfected with the empty pVAX I vector showed no detectable signal (Figure 1A). Concurrently, Western blot analysis of cell lysates revealed a single immunoreactive band specific to pVAX-GRA28 or pVAX-GRA83-transfected cells, respectively, which was absent in control groups (Figure 1B).

Furthermore, IL-28B expression was quantified in vitro using a commercial ELISA kit. Supernatants from pVAX-IL-28B-transfected cells contained elevated IL-28B levels, while those from empty-vector controls yielded no measurable expression (Figure 1C).

To confirm in vivo IL-28B expression, we quantified its serum levels by ELISA using blood samples collected from mice three days after the primary immunization with either pVAX-IL-28B or the empty pVAX1 vector. IL-28B was readily detectable in the sera of mice immunized with pVAX-IL-28B, whereas no expression was observed in the pVAX I vector control group (Figure 1D).

### 3.2. Humoral Immune Responses Induced by DNA Immunization

**Figure 2:**
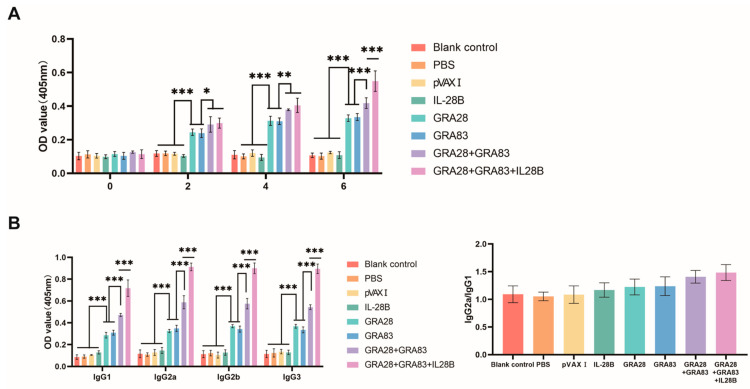
Analysis of *T. gondii*-specific humoral immunity elicited by single- and multi-antigen DNA vaccines. (**A**) Longitudinal monitoring of serum IgG levels in C57BL/6J mice immunized with pVAX-GRA28, pVAX-GRA83, or cocktail vaccines (weeks 0, 2, 4, 6). (**B**) IgG subclass profiling (IgG1, IgG2a, IgG2b and IgG3) at peak response (2 weeks post-final immunization). Statistical significance is indicated as *** *p* < 0.001, ** *p* < 0.01, * *p* < 0.05. Data are presented as means ± SD.

To evaluate humoral immunity, serum IgG and its subclasses (IgG1, IgG2a, IgG2b and IgG3) were quantified by ELISA at weeks 0, 2, 4, and 6 following immunization and prior to challenge. As demonstrated in Figure 2A, all vaccinated groups exhibited substantially elevated IgG titers that increased progressively with subsequent immunizations (*p* < 0.05). Antibody production showed a clear dose-dependent response: the triple combination (pVAX-IL-28B + pVAX-GRA28 + pVAX-GRA83) elicited the strongest response, followed by the dual-antigen group (pVAX-GRA28 + pVAX-GRA83), while single-antigen formulations generated more modest responses. In contrast, none of the control groups displayed significant antibody elevation throughout the study period (*p* > 0.05).

Consistent with total IgG levels, experimental groups exhibited significantly elevated titers of IgG1, IgG2a, IgG2b and IgG3, along with a higher IgG2a/IgG1 ratio, compared to controls (blank, PBS, and pVAX I). The dual-antigen formulation (pVAX-GRA28 + pVAX-GRA83) generated a more pronounced Th1-skewed response, including higher Th1 subclass antibody levels of IgG2a, IgG2b and IgG3 and higher IgG2a/IgG1 ratio than single-antigen immunization. This effect was further amplified by pVAX-IL-28B co-administration, which yielded the highest levels of IgG2a, IgG2b and IgG3 and IgG2a/IgG1 ratio among all groups (Figure 2B). No significant intergroup differences were detected in control animals (*p* > 0.05).

### 3.3. Cytokine Production and Cytokine-Related Transcription Factors

**Figure 3:**
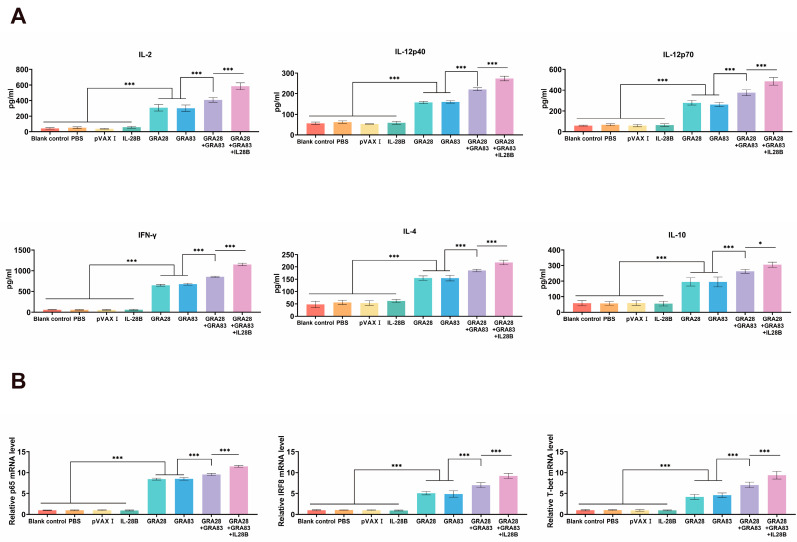
Polarization of cytokine responses and transcriptional regulators in vaccinated mice. (**A**) Cytokine secretion profiles (Th1/Th2) from splenocytes of mice immunized with single-antigen (pVAX-GRA28 or pVAX-GRA83), multi-antigen (pVAX-IL-28B + pVAX-GRA28) vaccines or cocktailed with pVAX-IL-28B + pVAX-GRA28 + pVAX-GRA83, measured by ELISA. (**B**) qRT-PCR analysis of key transcriptional regulators (p65/NF-κB, IRF8, T-bet) in splenocytes. Data shown as mean ± SD (* *p* < 0.05, *** *p* < 0.001).

Splenocytes were isolated two weeks post-immunization and cytokine production was assessed by ELISA after TLA stimulation. Multivalent DNA vaccination elicited significantly stronger Th1-type responses than single-gene immunization, as evidenced by elevated secretion of IFN-γ, IL-2, and IL-12 (both p40 and p70 subunits). The most pronounced cytokine response occurred in mice receiving the pVAX-IL-28B-adjuvanted cocktail vaccine. Parallel analysis revealed enhanced production of Th2-associated cytokines (IL-4 and IL-10) in all vaccinated groups relative to controls (Figure 3A). No significant intergroup differences were observed among control animals (*p* > 0.05).

Also, qRT-PCR quantification of immune-related transcription factors revealed distinct activation patterns in DNA-vaccinated mice relative to controls (Figure 3B). Specifically, we observed significant upregulation of key transcriptional regulators, including T-bet (controlling Th1 differentiation), IRF8 (modulating innate immunity), and p65/NF-κB (mediating inflammatory responses) (*p* < 0.05 for all). This transcriptional profile was consistently absent in all control groups (*p* > 0.05), demonstrating that pVAX-GRA28 and or pVAX-GRA83 vaccination selectively activates multiple arms of the immune response through these master regulators. Similarly, Co-administration with pVAX-IL-28B and pVAX-GRA28 + pVAX-GRA83 induced the most significantly upregulation of these key transcriptional regulators.

### 3.4. Cellular Immune Responses Induced by DNA Immunizations

**Figure 4:**
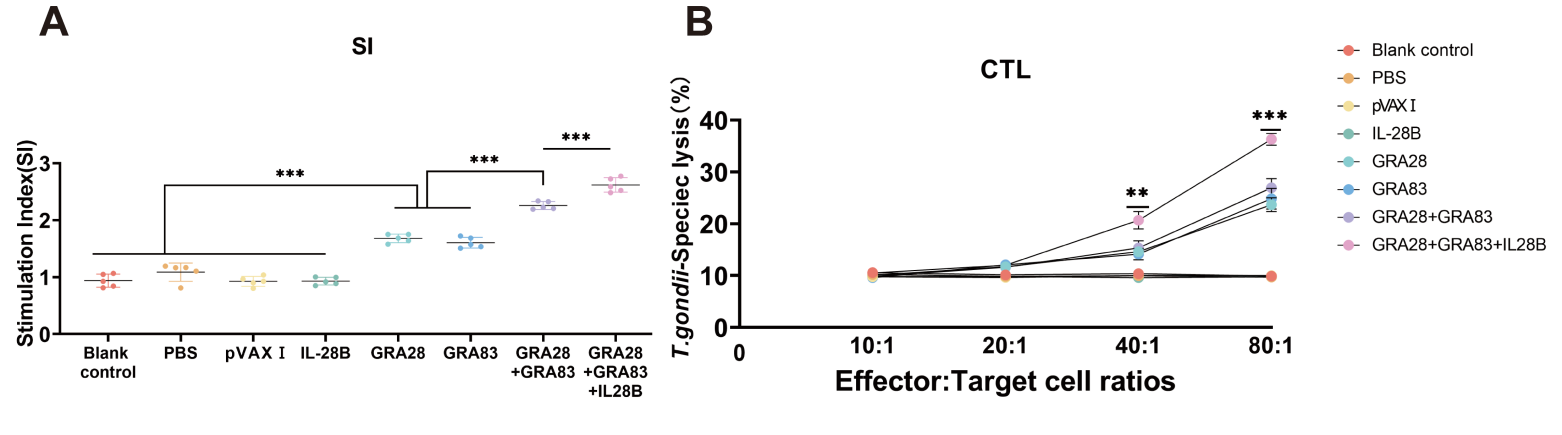
Cellular immune responses in vaccinated mice. (**A**) Splenocyte proliferative response in immunized and control mice. Lymphocyte proliferation stimulation index (SI) was calculated as [ratio of (OD570[TLA]/OD570[medium]): (OD570[ConA]/OD570[medium]] in immunized and control mice. (**B**) CTL activities of spleen lymphocytes in immunized mice. The effector-to-target cell ratios are indicated on the *x*–axis. The percentage of *T. gondii*-specific lysis is shown on the *y*–axis. *** *p* < 0.001. ** *p* < 0.01. Data are presented as the means ± SD.

Lymphocyte proliferation was evaluated by MTT assay following stimulation with TLA. Spleen cells from all vaccinated groups exhibited significantly higher stimulation indices (SI) than those from non-immunized controls (*p* < 0.05). Notably, co-administration of pVAX-IL-28B further potentiated the proliferative response in mice receiving the multi-gene DNA vaccine. In contrast, single-plasmid immunization groups showed comparable SI values (*p* > 0.05) (Figure 4A).

In addition, cytotoxic T lymphocyte (CTL) activity exhibited a dose-dependent enhancement with increasing effector-to-target ratios, reaching maximal lysis at an 80:1 ratio (Figure 4B). The combined pVAX-GRA28/pVAX-GRA83 immunization generated superior CTL responses compared to single-antigen formulations, while the pVAX-IL-28B-adjuvanted group demonstrated the most potent cytotoxicity. Control groups showed no statistically significant differences in CTL activity (*p* > 0.05).

### 3.5. DNA Vaccinations Activate Immune Responses Through the I-IFN Signaling Pathway

**Figure 5:**
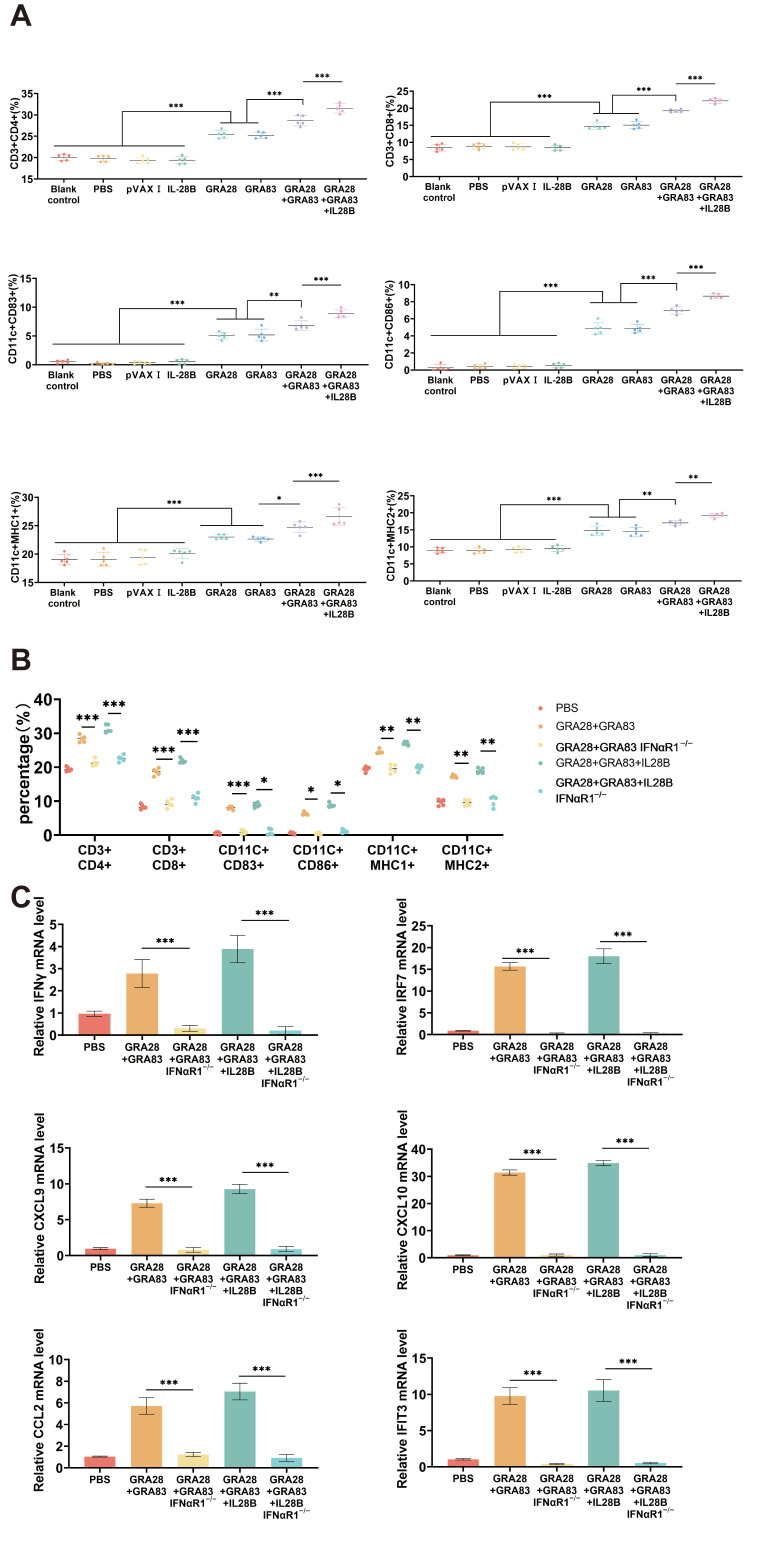
DNA vaccines activate immune responses through the I-IFN signaling pathway. C57BL/6J wild-type (WT) and IFNαR1−/− mice were DNA immunized with a single antigen (pVAX-GRA28 or pVAX-GRA83), multi-antigen (pVAX-IL-28B + pVAX-GRA28) vaccines or cocktailed vaccines with pVAX-IL-28B + pVAX-GRA28 + pVAX-GRA83. The mice were injected with PBS as a control group (*n* = 5 per group). Mouse splenic lymphocytes were isolated 14 days post the last immunization for flow cytometry analysis. (**A**,**B**) The numbers of CD4+, CD8+ T cells and DCs and the expressions of MHCI and MHCII in activated DCs in C57BL/6J wild-type (WT) (**A**) and IFNαR1−/− mice (**B**). (**C**) qPCR was used to detect the expression levels of interferon-stimulated genes (ISGs) in splenic lymphocytes of WT and IFNαR1−/− mice at 14 days post the last immunization. *** *p* < 0.001. ** *p* < 0.01. * *p* < 0.05. Data are presented as the means ± SD.

To decide the significance of the type I interferon (I-IFN) signaling pathway in DNA immunization, we investigated its role in DNA vaccination-mediated immune cell activation. Flow cytometric analysis of splenic lymphocytes 14 days post the last DNA immunizations revealed markedly attenuated immune cell activation in IFNαR1−/− mice compared to wild-type (WT) controls. This impairment was evidenced by significantly reduced numbers across multiple cell populations, including T cell subsets (CD4+ and CD8+ T cells), dendritic cells (DCs), and MHC I and MHC II expressions in DCs (Figure 5A,B).

Complementary qPCR analysis demonstrated concurrent downregulation of key I-IFN pathway components and interferon-stimulated genes (ISGs) in spleen of IFNαR1−/− mice, including IRF7, CXCL9, CXCL10, IFN-γ, CCL2, and IFIT3 (Figure 5C). These findings collectively demonstrate that DNA vaccination creates a robust yet transient immunostimulatory niche, with I-IFN signaling serving as a critical regulator of DNA vaccination-triggered immune activation.

### 3.6. IFNαR1 Knockout Impacts DNA Vaccination-Mediated Protective Efficacies in Mice

**Figure 6:**
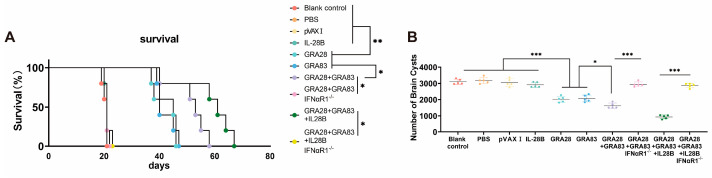
Protective efficacy of DNA vaccination against acute and chronic toxoplasmosis. (**A**) Survival kinetics of C57BL/6J and IFNαR1 knockout mice (*n* = 10/group) following oral challenge with 100 cysts of *T. gondii* ME49 strain at 2 weeks post-immunization. The triple-antigen IL-28B-adjuvanted vaccine (pVAX-IL-28B + pVAX-GRA28 + pVAX-GRA83) conferred the highest protection, with 80% survival at day 55, whereas all PBS control mice succumbed by day 21. (**B**) Chronic infection burden quantified as brain cyst counts in C57BL/6J and IFNαR1 knockout mice (*n* = 6/group) orally challenged with 10 cysts of the ME49 strain. Cysts were enumerated in whole-brain homogenates at 4 weeks post-challenge. Data are means ± SD. *** *p* < 0.001. ** *p* < 0.01. * *p* < 0.05. Data are presented as the means ± SD.

Type I interferons (I-IFNs) represent a multifunctional cytokine family that orchestrates both innate immune activation and adaptive immune polarization [26]. These cytokines enhance dendritic cell (DC) maturation and promote type 1 immune responses in T cells. To investigate the role of I-IFN signaling in IL-28B-adjuvanted DNA vaccines induced protective immunity in WT versus IFNαR1−/− mice following DNA immunization with pVAX-GRA28 + pVAX-83 plus pVAX-IL-28B. Then, the protective efficacy of vaccination was assessed through two challenge models. In the non-lethal challenge model (10 *T. gondii* ME49 cysts), cyst burden was quantified in brain tissue. For lethal challenge (100 *T. gondii* ME49), survival was monitored until endpoint. As demonstrated in Figure 6A.

In the lethal challenge model (100 *T. gondii* ME49 cysts), all vaccinated groups exhibited significantly prolonged survival compared with PBS controls (*p* < 0.05). Specifically, survival rates at 55 days post-challenge were 0% for mice immunized with pVAX-GRA28, 0% for pVAX-GRA83, 40% for the dual-antigen combination (pVAX-GRA28 + pVAX-GRA83), and 80% for the triple combination vaccine adjuvanted with IL-28B (pVAX-IL-28B + pVAX-GRA28 + pVAX-GRA83). In contrast, all PBS control mice succumbed within 21 days post-challenge, and the protective efficacies of IL-28B-adjuvanted DNA vaccines were not significantly affected in IFNαR1−/− mice compared to wild-type controls, suggesting that IL-28B–mediated adjuvant effects are independent of type I IFN signaling and instead likely mediated through type III IFN pathways. Notably, vaccination failed to confer comparable protection in IFNαR1−/− mice, in which survival rates were not significantly different from those observed in non-vaccinated WT controls. These findings indicate that type I IFN signaling is indispensable for the full protective efficacy of DNA vaccines.

Quantification of cerebral cyst burden revealed significant protection across all vaccinated groups (Figure 6B). Compared to controls, cyst reduction reached 39% (pVAX-GRA28), 34% (pVAX-GRA83), 54% (dual-antigen), and 81% (pVAX-IL-28B-adjuvanted triple combination). Control groups showed no statistically significant cyst reduction (*p* > 0.05). Similarly, there was not significant differences in cyst reduction in IL-28B-adjuvanted DNA vaccines in IFNαR1−/− mice compared to those in non-vaccinated WT mice.

## 4. Discussion

DNA vaccines have emerged as a promising platform for eliciting sustained protective immunity against *T. gondii* infection [30]. Building upon previous investigations of GRA antigen vaccines [31], we demonstrate that a bivalent DNA vaccine encoding TgGRA28 and TgGRA83 induces robust humoral and Th1-polarized cellular responses. This immunization strategy conferred dual protection, significantly improving survival rates against acute ME49 strain tachyzoites challenge while reducing cyst burden during chronic ME49 strain cysts infection. The superior efficacy of this bivalent formulation compared to single-antigen vaccines aligns with established principles of antigenic synergy observed in multi-epitope vaccination approaches [32,33]. Our findings reinforce the potential of cocktail DNA vaccines to generate comprehensive immunity against both acute and chronic toxoplasmosis, while highlighting TgGRA28/GRA83 as particularly promising protective antigens.

Adjuvants are essential components of modern vaccine formulations that enhance immunogenicity through three primary mechanisms: (1) prolonging antigen persistence at the inoculation site to sustain immune exposure, (2) facilitating targeted uptake by dendritic cells to improve antigen presentation, and (3) activating innate immune pathways to potentiate adaptive responses. These synergistic functions not only amplify the magnitude and durability of protective immunity but also enable significant antigen dose reduction—a critical advantage for vaccine development and deployment [34]. This study demonstrates that incorporating IL-28B as a molecular adjuvant significantly enhances the immunogenicity of a bivalent DNA vaccine (pVAX-GRA28/pVAX-GRA83) against toxoplasmosis. Co-delivery of pVAX-IL-28B potentiated multiple arms of adaptive immunity, including elevated antigen-specific antibody production, enhanced T cell proliferative capacity, as well as robust Th1-polarized cytokine responses and strengthened cytotoxic T lymphocyte activity. This comprehensive immune enhancement translated to superior protection against both acute and chronic *T. gondii* infection in murine models, establishing IL-28B as a promising adjuvant candidate for anti-parasitic vaccines, which is in line with some previous studies focused on the genetic adjuvant cytokines, such as pVAX-IL-36γ and pVAX-IL-33 [21,35]. However, further comparative studies are warranted to systematically evaluate the adjuvant efficacy of IL-28B relative to these immunomodulatory cytokines.

Our DNA immunization with pVAX-GRA28 and or pVAX-GRA83 establishes a balanced Th1/Th2 immune response critical for effective anti-*Toxoplasma* immunity. Vaccinated mice exhibited significantly elevated production of both Th1 cytokines (IFN-γ, IL-2 and IL-12) and Th2 cytokines (IL-4 and IL-10) compared to controls. Mechanistically, IL-12 drives IFN-γ production through MyD88-dependent signaling, initiating a cascade of parasiticidal effects including amino acid depletion (tryptophan via IDO, arginine via NOS2), IRG-mediated vacuole disruption, and inhibition of both tachyzoite proliferation and cyst reactivation [36,37]. Crucially, while the Th1 response mediates pathogen clearance, the concomitant Th2 response prevents lethal immunopathology—with IL-10 constraining excessive inflammation and IL-4 exhibiting temporal regulation [38]. This dual induction mirrors the protective efficacy of leading vaccine candidates (e.g., GRA24/MYR1 DNA vaccines) while overcoming the limitations of Th1-skewed formulations (e.g., GRA7 vaccine) that lack immunomodulatory capacity [39,40,41]. Collectively, these results demonstrate that DNA immunization with pVAX-GRA28 and or pVAX-GRA83 achieves the optimal cytokine balance required for comprehensive protection against toxoplasmosis.

The IFN-γ-mediated immune response against *T. gondii* involves a sophisticated regulatory network centered on three key molecular pathways: (1) The NF-κB pathway, where p65 (RelA) prevents parasite-induced apoptosis and is essential for infection control, as demonstrated by the susceptibility of p65 KO cells; (2) The IRF8-dependent pathway, which orchestrates IL-12 production through TLR11/MyD88 signaling and is critical for dendritic cell function; and (3) The T-bet axis, which directs Th1 differentiation and innate IFN-γ production that feeds back to sustain IRF8 activation [42,43,44]. Our vaccination studies revealed that DNA vaccination with pVAX-GRA28 and or pVAX-GRA83 significantly upregulated expression of these pivotal regulators (IRF8, T-bet, and p65), establishing a molecular signature consistent with robust protective immunity. These findings align with established mechanisms of IFN-γ induction through coordinated IRF8, T-bet, and NF-κB activation [45,46], confirming the vaccine’s ability to engage critical anti-parasitic defense pathways.

Mature DCs exhibited enhanced surface expression of both CD83—a critical mediator of inflammatory resolution [46]—and CD86, which provides essential co-stimulation through CD28 interaction to potentiate T cell responses [47]. The DNA vaccine formulations (pVAX-GRA28 and or pVAX-GRA83) demonstrate potent dendritic cell (DC) activation capacity, as evidenced by significant upregulation of key immunoregulatory markers. These findings align with established mechanisms of *T. gondii*-induced DC maturation [48] and confirm the vaccine’s ability to promote professional antigen presentation. Notably, immunized animals showed preferential MHC-II expression, indicative of robust CD4+ T cell priming potential, while maintaining detectable MHC-I presentation to support cytotoxic CD8+ T cell activation [49]. This balanced MHC profile suggests the vaccine optimally engages both major histocompatibility complex pathways to coordinate comprehensive adaptive immunity.

The host defense against *T. gondii* involves a coordinated interplay between CD4+ and CD8+T cell immunity. Our findings demonstrate that the DNA vaccine formulations effectively mobilize both arms of this protective response. Vaccination triggered significant expansion of CD4+ T lymphocytes, which orchestrate anti-parasitic defense through macrophage activation and cytokine-mediated recruitment of immune effectors to infection sites [50]. Concurrently, we observed robust CD8+ T cell differentiation into cytotoxic lymphocytes capable of direct parasite elimination through antigen-specific mechanisms [51]. The vaccine’s immunogenicity profile showed striking parallels with some other established nano-vaccine platforms, including ribosomal P2 [52] and TGGT1_278620 mRNA [46], all demonstrating similar enhancement of T lymphocyte populations. Notably, DNA immunization with pVAX-GRA28 and or pVAX-GRA83 elicited superior CTL activity compared to control groups, confirming its ability to generate potent cellular immunity against intracellular pathogens. This immunoprotective pattern closely resembles the efficacy demonstrated by leading DNA vaccine candidates employing ROP5 and ROP18 antigens [21], underscoring the vaccine’s potential as an effective anti-toxoplasmosis strategy.

The humoral immune response constitutes a vital defense mechanism against *T. gondii* infection, with B-cell mediated immunity playing a pivotal role in pathogen control [33]. Our studies reveal that DNA vaccination with pVAX-GRA28 and or pVAX-GRA83 elicit robust antibody production, particularly IgG isotypes that mediate protection through multiple effector mechanisms. These antibodies not only directly inhibit parasite replication but also facilitate macrophage-mediated clearance via antibody-dependent cellular cytotoxicity (ADCC) by enhancing parasite-opsonization and immune cell recognition [53]. Notably, the vaccine induced balanced yet Th1-skewed humoral immunity, as evidenced by significantly elevated levels of both IgG2a, IgG2b and IgG3 (Th1-associated) and IgG1 (Th2-associated) subtypes, with a markedly increased IgG2a/IgG1 ratio. This mixed but Th1-dominant antibody profile mirrors the protective immune signatures observed with several effective DNA vaccine candidates, including those targeting TgIST, TgNSM [35], GRA35, GRA42 and GRA43 [26] antigens. The coordinated induction of both Th1 and Th2 humoral responses suggests the vaccine’s capacity to mobilize comprehensive antibody-mediated defenses while maintaining the Th1 polarization crucial for optimal protection against intracellular pathogens.

While traditionally recognized for their antiviral properties, type I interferons (I-IFNs) exhibit complex immunomodulatory functions during bacterial infections, demonstrating both protective effects and potential to interfere with type II IFN-mediated defenses [54]. In the context of mRNA-based vaccination, preclinical studies consistently demonstrate I-IFN induction as a hallmark immune signature [55,56,57]. This response appears functionally significant, as evidenced by the MDA5-dependent I-IFN signaling required for BNT162b2-mediated CD8+ T cell activation [32] and the I-IFN-polarized immunity correlating with protective efficacy of influenza mRNA vaccines in non-human primates [58]. These findings collectively position I-IFN induction as a conserved mechanism underlying vaccine immunogenicity, particularly in generating cytotoxic T cell responses [59,60], while highlighting the pleiotropic nature of interferon biology across different pathogen contexts. Also, type I interferon (IFN-I) signaling plays a dual role in *T. gondii* infection, mediating critical host defense mechanisms in the central nervous system while paradoxically being subverted by the parasite to facilitate chronic persistence [61,62]. Although IFN-I is known to be essential for controlling established CNS infection, its potential role in vaccine-induced protective immunity against toxoplasmosis remains undefined. Specifically, whether anti-*Toxoplasma* vaccines can harness IFN-I pathways to initiate early innate immune responses capable of preventing infection or limiting parasite dissemination has yet to be systematically investigated. Our cytokine profiling revealed that DNA candidate administration potently activates type I interferon (I-IFN) signaling pathways, as demonstrated by upregulated expression of I-IFN genes (IFN-α, IFN-β), interferon-stimulated genes (ISG15, MX-1, IFIT3, IRF7), and key chemokines (CXCL9-11, CCL2-3). Emerging evidence positions I-IFN as a central regulator of adaptive immunity through dual mechanisms: (1) indirectly via enhanced antigen presentation through upregulated costimulatory molecules on antigen-presenting cells (APCs), and (2) directly through T cell stimulation and IFN-γ induction [63,64]. The critical role of I-IFN signaling in cellular immunity is further supported by studies showing that DC-specific IFNαR deficiency impairs CD8+ T cell cross-priming and tumor rejection [65]. Our findings extend this paradigm by demonstrating that DNA vaccines leverage I-IFN-mediated recruitment of cytotoxic T lymphocytes through ISG-derived chemotactic signals (CXCL9-11) [66], while likely cooperating with other immunomodulatory factors to achieve optimal CD8+ and CD4+ T cell responses. These results collectively establish I-IFN signaling as an essential component of DNA vaccine immunogenicity, operating through both direct and indirect immunostimulatory pathways. While IL-28B (IFN-λ3) primarily signals via the IFNLR1/IL10RB receptor complex, some degree of crosstalk with type I IFN pathways has been reported in other models. However, our findings indicate that the adjuvant effects of IL-28B are largely independent of IFNAR1-mediated signaling, consistent with canonical type III IFN activity.

## 5. Conclusions

Our findings demonstrate that DNA vaccines encoding GRA28, GRA83, or their combination elicit robust antigen-specific humoral immunity and Th1-polarized cellular responses, synergized with cytotoxic T lymphocyte (CTL) activity, conferring protection against both acute and chronic *T. gondii* infection in murine models. Notably, co-administration of the genetic adjuvant IL-28B significantly potentiated vaccine-induced adaptive immunity, enhancing both the magnitude and durability of protective responses by activating the I-IFN response. These results not only advance the development of an effective anti-toxoplasmosis vaccine but also provide a promising strategy for immunization against other apicomplexan parasites.

## Figures and Tables

**Table 1:** The primers used for RT-PCR amplification of the NF-κB p65, T-bet, and β-actin genes were designed using DNASTAR software (version 11.1.0.54; Madison, WI, USA).

Primer Name	Sequence
B-Actin-F	5′-GCTTCTAGGCGGACTGTTAC-3′
B-Actin-R	5′-CCATGCCAATGTTGTCTCTT-3′
NF-KB p65-F	5′-GAACCAGGGTGTGTCCATGT-3′
NF-KB p65-R	5′-TCCGCAATGGAGGAGAAGTC-3′
T-bet-F	5′-GCCAGGGAACCGCTTATATG-3′
T-bet-R	5′-TGGAGAGACTGCAGGACGAT-3′

## Data Availability

The original contributions presented in this study are included in the article. Further inquiries can be directed to the corresponding author.
